# *TCAP* gene is not a common cause of cardiomyopathy in Iranian patients

**DOI:** 10.1186/s40001-023-01019-4

**Published:** 2023-09-26

**Authors:** Zahra Alaei, Nasrin Zamani, Bahareh Rabbani, Nejat Mahdieh

**Affiliations:** 1 Faculty of Basic Sciences, Islamic Azad University, East Tehran Branch, Tehran, Iran; 2 Cardiogenetic Research Center, Rajaie Cardiovascular Medical and Research Center, Iran University of Medical Sciences, Tehran, Iran; 3 Growth and Development Research Center, Tehran University of Medical Sciences, Tehran, Iran; 4 Genetics Laboratory, Rajaie Cardiovascular Medical and Research Center, Vali-E-Asr Avenue, Tehran, 1996911151 Iran

**Keywords:** Cardiomyopathy, Hypertrophic cardiomyopathy, Dilated cardiomyopathy, TCAP gene

## Abstract

**Background:**

Hypertrophic cardiomyopathy (HCM) and dilated cardiomyopathy (DCM) are the most frequent cardiomyopathies that cause acute heart failure and sudden cardiac death. Previous genetic reports have shown that pathogenic variants of genes encoding Z-disc components such as telethonin protein (TCAP) are the primary cause of DCM and HCM.

**Methods:**

This study was the first investigation on the TCAP gene among the Iranian cardiomyopathies population wherein the *TCAP* gene was analyzed in 40 unrelated patients (17 females and 23 males) who were clinically diagnosed with HCM and DCM. In addition, we conducted a thorough review of all published articles and the databases that were the first to report novel pathogenic or likely pathogenic variants the in *TCAP* gene.

**Results:**

In the cohort of this study, we identified only one intronic variant c.111-42G > A in one of the HCM patients that were predicted as polymorphism by in-silico analysis. Moreover, a total of 44 variants were reported for the *TCAP* gene in the literature where a majority of mutations were found to be missense. Pathogenic mutations in *TCAP* may cause diseases including limb-girdle muscular dystrophy 2G (LGMD-2G), DCM, HCM, intestinal pseudo-obstruction, and telethonin deficiency. However, a large number of affected patients were clinically diagnosed with limb-girdle 2G compared to other presenting phenotypes.

**Discussion:**

These findings suggest that the *TCAP* gene pathogenic mutations might not be a common cause of cardiomyopathies among Iranian patients. These gene disease-causing mutations may cause various manifestations, but it has a high prevalence among LGMD-2G, HCM, and DCM patients.

## Introduction

Cardiomyopathies are defined as a heterogeneous group of pathological conditions stemming from the myocardium's electrical and/or mechanical dysfunction [1]. *T*hese disorders can be categorized into primary cardiomyopathies which are due to genetics, acquired or mixed factors and solely affect the heart, and secondary cardiomyopathies which result from a systemic illness and impact several parts of the body causing different manifestations including hypertrophic (HCM), dilated (DCM) and restrictive (RCM) and arrhythmogenic right ventricular cardiomyopathy (ARVC) [1, 2]. Hypertrophic cardiomyopathy (HCM) is the most prevalent genetic myocardial disease caused by left ventricular hypertrophy which can cause exertional dyspnea, presyncope, atypical chest pain, heart failure, and sudden cardiac death [3]. Dilated cardiomyopathy (DCM) can be either genetic or acquired heart disease, characterized by left ventricular dilation and systolic dysfunction. DCM typically manifests symptoms of heart failure with reduced ejection fraction and in severe cases requires cardiac transplantation [1, 3]. However, restrictive cardiomyopathy (RCM) is less frequent and commonly associated with impaired ventricular filling with biatrial enlargement but with normal or decreased diastolic volume in one or both ventricles [4].

After the identification of pathogenic variants in the myosin heavy chain 7 (*MYH7*) gene and the cardiac alpha-actin (*ACTC1*) gene in developing HCM and DCM, respectively, over 100 genes have been reported to cause cardiomyopathies [3]. For instance, it has been found that mutations in genes, such as *CSRP3 (MLP protein)*, *TTN* (titin protein), and *TCAP* (telethonin protein) encode several Z-disc proteins of the cardiac sarcomere can lead to several cardiac dysfunctions [5, 6]. TCAP-encoded telethonin is a genetic determinant that implicates the pathogenesis of both DCM and HCM which is required for the structural organization of sarcomere assembly and acting as a stretch sensor, regulates the sarcomere length [3, 7]. Therefore, molecular studies on Z-discs mutations showed that pathogenic variants disturbing the function of the telethonin protein can lead to cardiac complications and severe myocyte hypertrophy [6, 8]. Of interest is that a group of HCM-associated TCAP mutations increases binding to other cardiac proteins, such as titin/connectin and calsarcin-1. These variants augment the interaction of TCAP with titin and CS-1 in the Z-disc. This augmentation may employ an increased passive tension which might lead to elevated calcium sensitivity in muscle contraction at the constant length of muscle fiber [9]. On the other hand, any disturbance in proteins that closely interact with telethonin (TCAP) may derange the myocardium function. For instance, the telethonin (TCAP) was found to bind to a cell surface protein BMP10 at the stretch-sensing Z disc of cardiomyocytes. TCAP partially regulates prohypertrophic BMP10, thereby pathogenic variants in the BMP10 gene deter binding to TCAP and increase dilated cardiomyopathy occurrence [10]. *Some patients* harbored pathogenic mutations of MLP/TCAP-HCM that phenotypically resemble myofilament-HCM and experience more severe conditions than the subset of patients who remain without a disease-causing mutation [5]. Similarly, DCM-associated MLP mutations reduce binding to TCAP/telethonin and actinin proteins whereas DCM-associated alpha-actinin-2 mutation reduces binding to MLP. These observations indicate that changed interaction in Z-disc components triggers cardiomyopathy, more specifically reduced binding interaction might cause loose sarcomere and decrease the stretch response of cardiomyocytes, while increased binding and stiff sarcomere, may become highly susceptible to the hypertrophic response of cardiomyocytes against stretch [11].

In this study, we investigate the frequency, clinical phenotypes, and spectrum of TCAP genetic variants in a cohort of hypertrophic cardiomyopathy (HCM) and dilated cardiomyopathy (DCM) patients referring to our tertiary center in Tehran. The disease-causing effects and functional consequences of the variants in the M-domain of the Titin protein were also determined using prediction analysis.

## Materials and methods

### Sample collection

A total number of 74 patients diagnosed with hypertrophic (HCM) and dilated (DCM) cardiomyopathies were registered in the cohort of this study, these patients were referred to our center between 1959 and 2018. Informed consent was obtained from all of the patients. Clinical data were documented from the medical records and provided questionnaires. The given questionnaires thoroughly assembled the information regarding the clinical presentations, other diseases, syndromes, and environmental factors of each patient. The local ethics committee of the Cardiovascular, Medical Research Center approved the research protocol by protocol number (no IR.IUMS.REC.1399.157).

### Clinical evaluations

Medical evaluations were performed and family histories were recorded for further analysis. From the cohort of this study, 40 patients were selected for genetic testing from whom 20 individuals were diagnosed with HCM (7 females and 13 males), and the other 20 patients were diagnosed with DCM (10 females and 10 males). Clinical presentations in most cases include hypertension, palpitation, dyspnea, syncope, fatigue, and high blood lipid, even though a handful of patients presented cardiac and abdominal edema, ischemia, kidney, and chest pain, muscular complications, and liver disorder. The diagnosis of HCM and DCM in all the patients of this study were confirmed by specialized physicians and the findings of medical testing were Echocardiography (ECG), Magnetic Resonance Imaging (MRI), two-dimensional echocardiography, and necessary clinical laboratory tests.

### Molecular analysis

5 ml of peripheral blood sample was obtained from each patient for genetic analysis. Genomic DNA was extracted based on the salting-out procedure. Primers were designed for coding regions which include exon and exon-intronic boundaries of the TCAP gene (NM_003673) (Table 1), and the coding regions were amplified by a SimpliAmp™ Thermal Cycler.

**Table 1:** Reverse and forward primers used in this study for PCR procedure

Exon No.	Primer sequences 5′ to 3′ direction	Primer length	Product length	GC%	TM(˚c)
1 Forward	ACTTATAGCATCTGACACCAGAGG	24 bp	956	45.8	69.6
1 Reverse	AAATTTCTCCAGGGCTTCATG	21 bp	956	42.9	72.1
2 Forward	TGAAGCCCTGGAGAAATTTCTG	22 bp	956	45.5	74.1
2 Reverse	GCAAACTACAAAGCAGCCATG	20 bp	956	47.6	72.2

Then, PCR was performed in a volume of 50 μL reagents on the following condition: 200 ng DNA, 1.5–2 mmol/L MgCl2, 10.5 μL primers (Forward and Reverse primers), 200 mmol/L dNTP, and 1 U of Taq DNA polymerase. The PCR thermal program was initial denaturation for 5 min at 95 °C and 30 cycles for denaturation at 95 °C (30 s), annealing at 58 °C (30 s), extension at 72 °C (30 s), and final extension at 72 °C (10 min). Direct sequencing was implemented with the BigDye Terminator DNA sequencing kit and Genetic Analyzer (Applied Biosystems, Foster City, CA, USA). Segregation analysis was performed for the families who had a novel variant.

The cohort was examined only for TCAP gene variants, since it had never been investigated in the Iranian population. All the mutations identified in the 40 patients of this study’s cohort are shown in Tables 3 and 4.

### In Silico analysis

Pathogenicity and clinical significance of *TCAP* gene variants were analyzed using bioinformatic predicting software, such as MutationTaster, Provean, SIFT, and CADD [12–15]. Nucleotide and protein change, zygosity, and the main clinical manifestations and symptoms were included in the investigation in Tables 1 and 2.

**Table 2:** General information and clinical presentations of the cohort participated in this study

Patient No.	Sex	Age	symptom	Final results of Echocardiogram (ECG)							
				IVSD (cm)	PWd (cm)	LVIDs (cm)	LVIDd (cm)	LVEF (%)	LAD (cm)	LVOTO ±	SAM ±
1	F	60	Hypertension/palpitations	2.1	1	2.8	4.4	60	4	–	–
2	F	32	Palpitations/dyspnea/fatigue/chest pain/syncope/anemia	1.1	0.9	–	4.4	40–45	–	–	–
3	M	16	Palpitations/dyspnea/fatigue/chest pain/gastrointestinal disorder/high cholesterol/allergy	3.2	0.9	3.45	4.29	60	3	–	+
4	M	58	Chest pain/hypertension/high cholesterol/muscular pain	1.6	0.86	3.9	5.3	20	4.5	N/A	N/A
5	F	43	Palpitations/dyspnea/abdominal edema/ischemia	1.5	0.91	3.52	4.65	15–20	5.2	–	–
6	M	8	Hypertension/palpitations	2.2	1	2.6	4.4	40–45	4.1	–	–
7	F	28	Fatigue/palpitations/hypertension	2.2	N/A	N/A	N/A	45	N/A	N/A	–
8	F	30	Hypertension/dyspnea/fatigue	1.8	0.7	2.5	4.3	45–50	3.1	N/A	–
9	M	26	Chest pain/dyspnea	0.9	0.8	5.8	6.8	10	4.1	–	–
10	F	7	Musculoskeletal disorder/Infectious disease	N/A	N/A	N/A	N/A	10–15	N/A	N/A	N/A
11	F	10	Dyspnea/palpitations/kidney problem	N/A	N/A	5.6	6.2	10	N/A	N/A	N/A
12	M	37	Cardiovascular disease/respiratory disease	0.8	0.8	5.8	6.4	10	N/A	N/A	N/A
13	M	24	Hypertension/high blood lipid/high triglyceride/fatigue/palpitations/dyspnea/fainting	0.9	0.9	6.1	7.7	15	4.7	N/A	N/A
14	M	2	Dyspnea/fatigue/chest pain/hypertension/liver disease/cardiac edema	1.5	N/A	N/A	N/A	55	N/A	N/A	N/A

*F* Female, *M* Male, *IVSD* Interventricular Septal thickness, *PWd* Posterior Wall thickness, *LVIDs* Left Ventricular Internal Diameter systole, *LVIDd* Left Ventricular Internal Diameter diastole, *LVEF* Left Ventricular Ejection Fraction, *LAD* Left Atrium Dimension, *LVOTO* Left Ventricular Outflow Tract Obstruction; SAM: Systolic Anterior Motion of the Mitral valve

### Data extraction

For extracting the necessary information associated with each article, a checklist was used to cover the mutation, mutation type, article title, first author's name, year of publication, ethnicity, sample size (total, men, and women), and the rate of consanguineous marriages.

### Genetic variant distribution

The variant position in protein was determined based on UniProtKB/SwissProt—O15273 and also using NM_003673. The Human Gene Mutation Database (HGMD) and reference SNP ID (rsID) for the variants were examined using the databases MutationTaster (https://mutationtaster.org/) and Varsome (https://varsome.com/), and if required, they were corrected. In addition, variants that were reported as pathogenic or likely pathogenic in ClinVar, but were not available in the literature were included in this study. Thereafter, the reported variants were divided into six types including missense, nonsense, deletion, insertion, intronic, and duplication.

## Results

### Clinical features

**Fig. 1:**
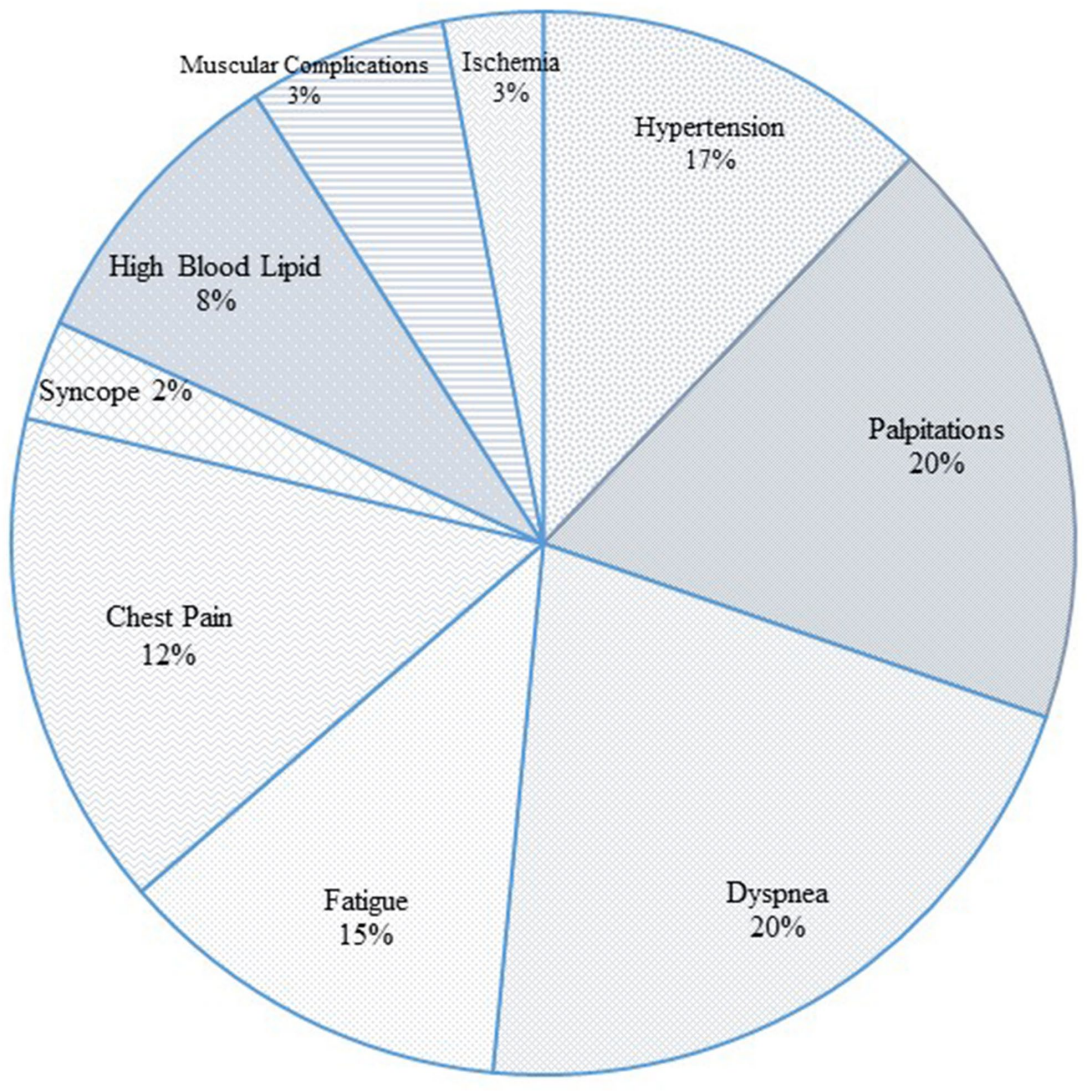
Number of patients who were clinically reported to have to these symptoms

Of the 40 patients in this study, half of them were diagnosed with HCM (10 females and 10 males) and the other half with DCM (13 males and 7 females). According to the statistical findings, the prevalence of DCM in this population is higher among males than females. The cohort is classified into three main age groups; children and youngsters (< 20), adults (40–20), middle-aged, and elderly (> 40). Medical history revealed that the symptoms started to manifest mostly in adulthood (44%) and middle-aged (35%) and a minority in childhood and youth (21%). On clinical examination, 7.69% of the patients with familial cardiomyopathy were shown to be asymptomatic whereas other patients had the most common presentations of hypertrophic or dilated cardiomyopathy, including hypertension, dyspnea, fatigue, chest pain, and palpitations (Fig. 1). Less common manifestations were recorded as fatigue, dizziness, and syncope. In addition, a few patients suffered from related severe conditions of myocardial infarction (7%,) and ischemic (3%). Some of the patients had received medical treatments which includes undergoing cardiac transplantation or ICD (32%) and angiography operation (10%). Several patients exhibited other complications, such as high cholesterol (24%), high blood pressure (19%), diabetes, and high blood sugar (8%). Considering environmental factors, some patients engaged in actions aggravating their conditions, such as smoking tobacco and consuming alcohol (17.56%) and being obese (2.7%). Only 16.21% of patients had daily physical activities. The clinical phenotypes and medical records of the patients are summarized in Table 2.

### Family history

By examining the information extracted from the questionnaires and genetic counseling of the patients, 12% of them were the only ones affected with cardiomyopathy in their family, while the rest of the cohort exhibited family histories of cardiomyopathy that involved 1 to 10 family members with heart disease and other related disorders. Several affected families (59% of patients’ families) showed cardiomyopathy transmission through up to three consecutive generations.

Following cardiac disorders, the second and third most commonly reported diseases among the family members of these patients were cancer (20%) and stroke (14%).

### Genetic analysis and bioinformatics findings

**Fig. 2:**
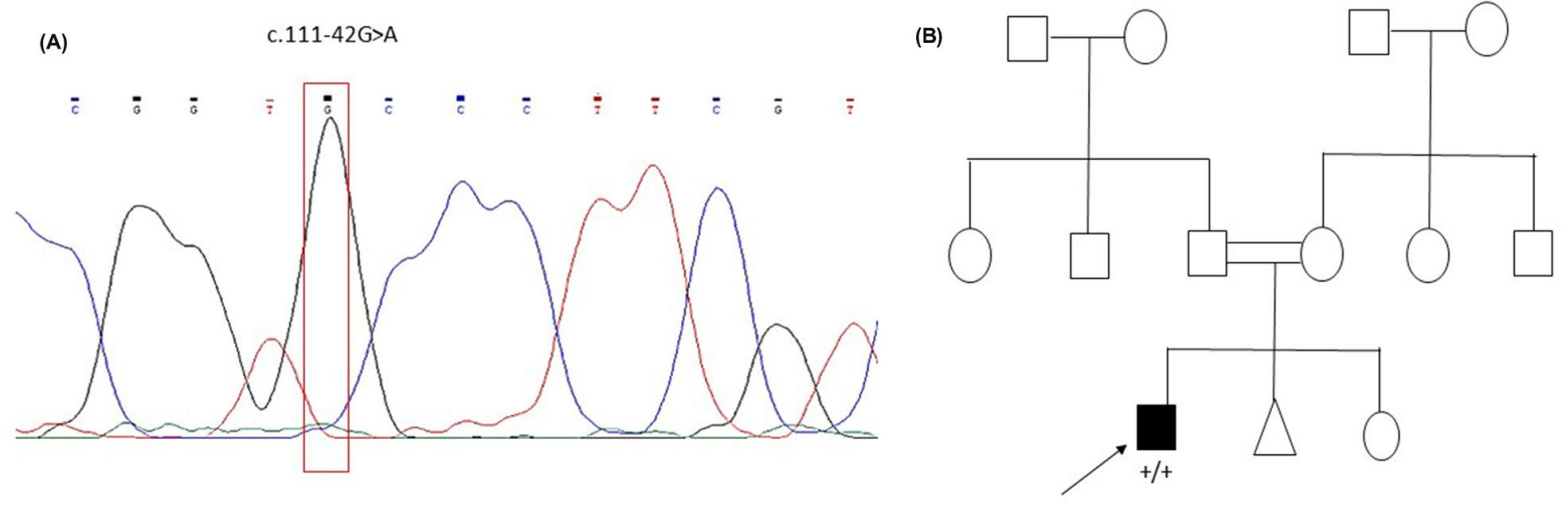
**A** Location of polymorphism variant on TCAP, **B** pedigree of the DCM patient who had the novel polymorphism variant

Our genetic analysis of the *TCAP* gene in the studied cohort revealed one novel intronic variant of c.111-42G > A in intron 1 of an HCM patient (No. 13). In silico analysis tools including MutationTaster, and CADD predicted this variant to be considered as polymorphism. In addition, Varsome considered this variant to have uncertain significance. The electropherogram (sequencing result) of the polymorphism variant and the pedigree of the patient are shown in Fig. 2.

As for other detected variants, 80% of DCM patients (accounted 18 out of 20) were found to possess previously reported variants of cDNA Level: NM_003673.3(TCAP): c.453A > C, gDNA Level: g.39666058A > C, Protein Level: (p.Ala151 =)(A151 =) whereas no mutation was detected in the remaining patients (20%). Furthermore, 70% of HCM patients (accounted 14 out of 20) were identified to have c.453A > C as well as two other patients with reported mutations including cDNA level: NM_003673.4(TCAP):c.316C > T, gDNA level: g.39665921C > T, protein level: (p.Arg106Cys)(R106C) and cDNA level: NM_003673.4(TCAP):c.110 + 48C > T gDNA level: g.39665517C > T. All the variants detected in this study’s cohort are shown on the *TCAP* gene depicted in Tables 3 and 4.

**Table 3:** Variants detected in DCM patients who participated in our study and in-silico prediction for each mutation

No.	Patient No	Nucleotide change	Amino Acid Change	Location	Zygosity	rs	Varsome	MutationTaster	Provean	SIFT	CADD	References
1	DCM1	No mutation found	–	–	–	–	–	–	–	–	–	–
2	DCM2	c.453A > C	p.Ala151 =	Exon 2	Homo	rs1053651	Benign	polymorphism	Neutral	Tolerated	10.92	[16]
3	DCM3	No mutation found	–	–	–	–	–	–	–	–	–	–
4	DCM4	c.453A > C	p.Ala151 =	Exon 2	Homo	rs1053651	Benign	polymorphism	Neutral	Tolerated	10.92	[16]
5	DCM5	c.453A > C	p.Ala151 =	Exon 2	Het	rs1053651	Benign	polymorphism	Neutral	Tolerated	10.92	[16]
6	DCM6	No mutation found	–	–	–	–	–	–	–	–	–	–
7	DCM7	c.453A > C	p.Ala151 =	Exon 2	Homo	rs1053651	Benign	polymorphism	Neutral	Tolerated	10.92	[16]
8	DCM8	c.453A > C	p.Ala151 =	Exon 2	Homo	rs1053651	Benign	polymorphism	Neutral	Tolerated	10.92	[16]
9	DCM9	c.453A > C	p.Ala151 =	Exon 2	Homo	rs1053651	Benign	polymorphism	Neutral	Tolerated	10.92	[16]
10	DCM10	c.453A > C	p.Ala151 =	Exon 2	Homo	rs1053651	Benign	polymorphism	Neutral	Tolerated	10.92	[16]
11	DCM11	c.453A > C	p.Ala151 =	Exon 2	Homo	rs1053651	Benign	polymorphism	Neutral	Tolerated	10.92	[16]
12	DCM12	c.453A > C	p.Ala151 =	Exon 2	Homo	rs1053651	Benign	polymorphism	Neutral	Tolerated	10.92	[16]
13	DCM13	c.453A > C	p.Ala151 =	Exon 2	Homo	rs1053651	Benign	polymorphism	Neutral	Tolerated	10.92	[16]
14	DCM14	c.453A > C	p.Ala151 =	Exon 2	Homo	rs1053651	Benign	polymorphism	Neutral	Tolerated	10.92	[16]
15	DCM15	c.453A > C	p.Ala151 =	Exon 2	Homo	rs1053651	Benign	polymorphism	Neutral	Tolerated	10.92	[16]
16	DCM16	c.453A > C	p.Ala151 =	Exon 2	Homo	rs1053651	Benign	polymorphism	Neutral	Tolerated	10.92	[16]
17	DCM17	c.453A > C	p.Ala151 =	Exon 2	Homo	rs1053651	Benign	polymorphism	Neutral	Tolerated	10.92	[16]
18	DCM18	No mutation found	–	–	–	–	–	–	–	–	–	–
19	DCM19	No mutation found	–	–	–	–	–	–	–	–	–	–
20	DCM20	No mutation found	–	–	–	–	–	–	–	–	–	–

**Table 4:** Variants found in HCM patients who participated in our study and in-silico prediction for each mutation

No.	Patient No.	Nucleotide change	Amino Acid Change	Location	Zygosity	rs	Varsome	MutationTaster	Provean	SIFT	CADD	References
1	HCM1	c.453A > C	p.Ala151 =	Exon 2	Het	rs1053651	Benign	polymorphism	Neutral	Tolerated	10.92	[16]
2	HCM2	c.316C > T	p.Arg106Cys	Exon 1	Het	rs45578741	Benign	polymorphism	Deleterious	Damaging	28.8	[17]
		c.453A > C	p.Ala151 =	Exon 2	Het	rs1053651	Benign	polymorphism	Neutral	Tolerated	10.92	[16]
3	HCM3	No mutation found	–	–	–	–	–	–	–	–	–	–
4	HCM4	No mutation found	–	–	–	–	–	–	–	–	–	–
5	HCM5	c.453A > C	p.Ala151 =	Exon 2	Het	rs1053651	Benign	polymorphism	Neutral	Tolerated	10.92	[16]
6	HCM6	c.453A > C	p.Ala151 =	Exon 2	Homo	rs1053651	Benign	polymorphism	Neutral	Tolerated	10.92	[18]
7	HCM7	c.453A > C	p.Ala151 =	Exon 2	Het	rs1053651	Benign	polymorphism	Neutral	Tolerated	10.92	[18]
8	HCM8	c.453A > C	p.Ala151 =	Exon 2	Homo	rs1053651	Benign	polymorphism	Neutral	Tolerated	10.92	[18]
		c.110 + 48C > T	–	Intron 1	Het	rs2941510	Benign	polymorphism	–	–	6.243	[18]
9	HCM9	No mutation found	–	–	–	–	–	–	–	–	–	–
10	HCM10	c.453A > C	p.Ala151 =	Exon 2	Homo	rs1053651	Benign	polymorphism	Neutral	Tolerated	10.92	[16]
11	HCM11	c.453A > C	p.Ala151 =	Exon 2	Homo	rs1053651	Benign	polymorphism	Neutral	Tolerated	10.92	[16]
12	HCM12	No mutation found	–	–	–	–	–	–	–	–	–	–
13	HCM13	c.453A > C	p.Ala151 =	Exon 2	Homo	rs1053651	Benign	polymorphism	Neutral	Tolerated	10.92	[16]
		c.111–42G > A	–	Intron 1	Homo	–	Uncertain Significance	polymorphism	–	–	**3.473**	This study
14	HCM14	c.453A > C	p.Ala151 =	Exon 2	Homo	rs1053651	Benign	polymorphism	Neutral	Tolerated	10.92	[16]
15	HCM15	c.453A > C	p.Ala151 =	Exon 2	Homo	rs1053651	Benign	polymorphism	Neutral	Tolerated	10.92	[16]
16	HCM16	c.453A > C	p.Ala151 =	Exon 2	Het	rs1053651	Benign	polymorphism	Neutral	Tolerated	10.92	[16]
17	HCM17	c.453A > C	p.Ala151 =	Exon 2	Het	rs1053651	Benign	polymorphism	Neutral	Tolerated	10.92	[16]
18	HCM18	c.453A > C	p.Ala151 =	Exon 2	Het	rs1053651	Benign	polymorphism	Neutral	Tolerated	10.92	[16]
19	HCM19	No mutation found	–	–	–	–	–	–	–	–	–	–
20	HCM20	No mutation found	–	–	–	–	–	–	–	–	–	–

### Genetic variant distribution and genotype

**Fig. 3:**
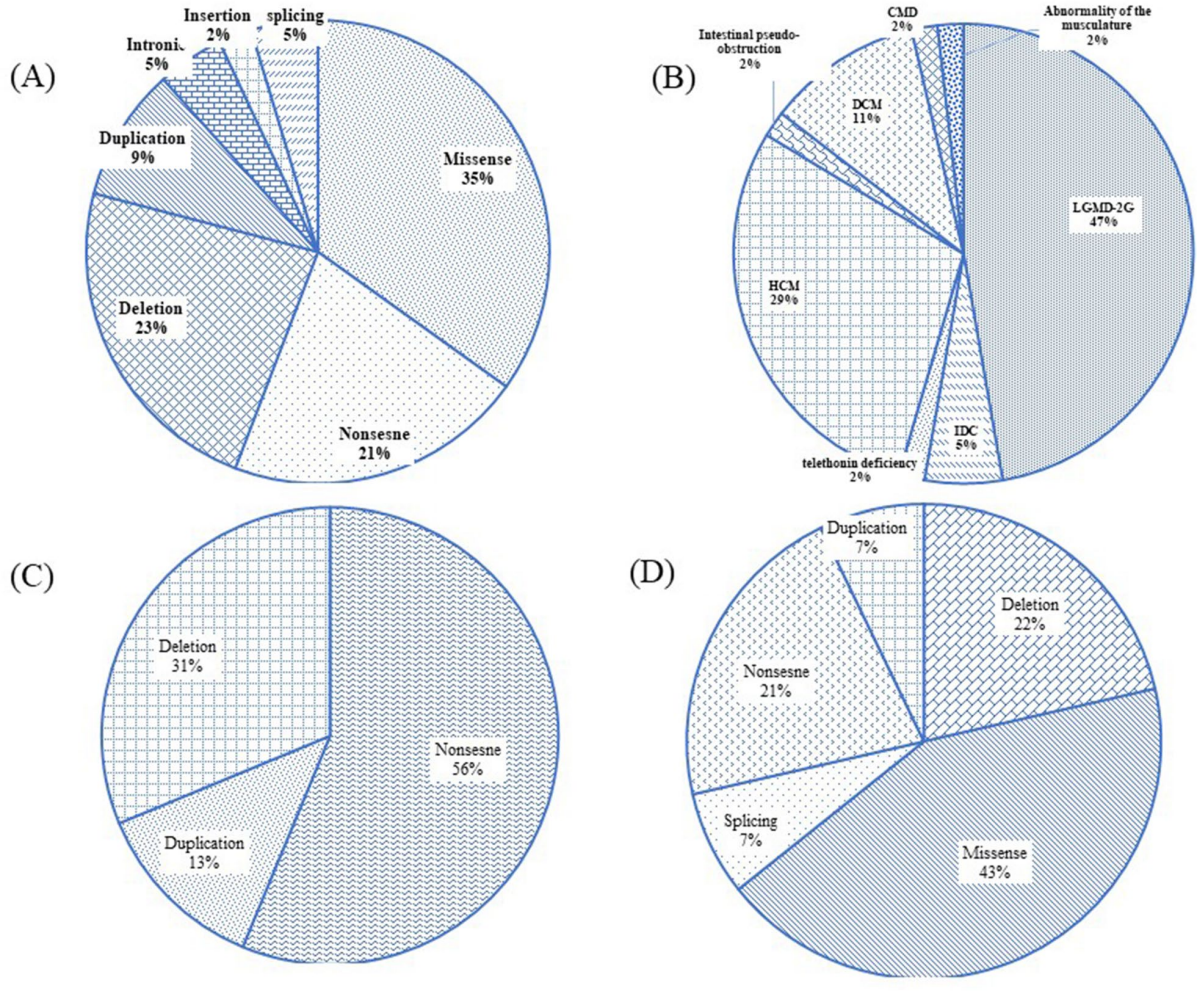
**A** Pie chart indicates TCAP gene mutation types reported in the literature and ClinVar, **B** chart presents the most common phenotype among patients, **C**, **D** chart shows the frequency of each TCAP variant types among the LGMD-2G and HCM patients, respectively

Variants associated with the *TCAP* gene in HGMD and Clinvar were extracted that have been identified thus far. A total of 44 reported mutations were included in Table 5. The three most common variants found to be missense, deletion, and nonsense make up 35% (15 alleles), 21% (10 alleles), and 21% (9 alleles) of the total variants (Fig. 3).

**Table 5:** List of variants of TCAP gene reported in Literature and databases

No.	DNA Change	AA Change	Variant Type	Location	RS
1	c.-178G > T		Splicing	5' UTR	rs931992
2	c.32C > A	p.Ser11Ter	Nonsense	Exon 1	rs45495192
3	c.453A > C	p.Ala151 =	Synonymous	Exon 2	rs1053651
4	c.53G > A	p.Arg18Gln	Missense	Exon 1	rs45614536
5	c.145G > A	p.Glu49Lys	Missense	Exon 2	rs45513698
6	c.421C > G	p.Pro141Ala	Missense	Exon 2	rs45509691
7	c.75G > A	p.Trp25Ter	Nonsense	Exon 1	rs778851652
8	c.157C > T	p.Gln53Ter	Nonsense	Exon 2	rs104894655
9	c.637–640delGG		Deletion	Exon 2	–
10	c.172C > T	p.Gln58Ter	Nonsense	Exon 2	–
11	c.37–39delGAG	p.Glu13del	Deletion	Exon 1	–
12	c.208C > T	p.Arg70Trp	Missense	Exon 2	rs775636212
13	c.269C > T	p.Pro90Leu	Missense	Exon 2	rs727504427
14	c. 226C > T	p.Arg76Cys	Missense	Exon 2	rs572836774
15	c.244C > T	p.Gln82Ter	Nonsense	Exon 2	–
16	c.255C > A	p.Tyr85Ter	Nonsense	Exon 2	–
17	c.316C > T	p.Arg106Cys	Missense	Exon 2	rs45578741
18	c.388C > T	p.Arg130Cys	Missense	Exon 2	rs374886575
19	c.472C > A	p.Arg158Ser	Missense	Exon 2	rs397516863
20	c.493C > G	p.Gln165Glu	Missense	Exon 2	rs397516865
21	c.410C > T	*p.*Thr137Ile	Missense	Exon 2	rs773317399
22	c.458G > A	p.Arg153His	Missense	Exon 2	rs149585781
23	c.395A > C	p.Glu132Gln	Missense	Exon 2	–
24	c.472C > T	p.Arg158Cys	Missense	Exon 2	rs397516863
25	c.90–91del	p.Ser31HisfsX11	Deletion	Exon 1	rs1555606976
26	c.26–33dupAGGTGTCG	p.Arg12fsX31	Duplication	Exon 1	rs778568339
27	c.45–46delTG	p.Cys15Ter	Deletion	Exon 1	–
28	c.100delC	p.Glu35Argfs*33	Deletion	Exon 1	–
29	c.166insG	p.Gln56Argfs*52	Insertion	Exon 2	–
30	c.496–499delAGAG	p.Arg166AlafsTer21	Deletion	Exon 2	–
31	c.171C > G	p.Cys57Trp	Missense	Exon 2	rs369447207
32	c.109–110delGG	p.Gly37Leufs	Deletion	Exon 1	–
33	c.110 + 5G > A		Intronic	Intron 1	–
34	c.25–31dup	p.Ser11Ter	Duplication	Exon 1	rs863224933
35	c.66G > A	p.Trp22Ter	Nonsense	Exon 1	rs141019458
36	c.*76G > T		Splicing	3’UTR	rs45506294
37	c.34dup	p.Glu12fs	Duplication	Exon 1	rs1555606959
48	c.43–49dup	p.Arg17delinsLeuTer	Duplication	Exon 1	rs886044421
39	c.103G > T	p.Glu35Ter	Nonsense	Exon 1	rs779699520
40	c.110_110 + 1del		Deletion	Exon 1	rs786205076
41	c.136_137del	p.Gln46Glufs*3	Deletion	Exon 2	rs2057249899
42	c.166C > T	p.Gln56Ter	Nonsense	Exon 2	–
43	c.110 + 1G > A		Intronic	Intron 1	–
44	c.14–15del	p.Glu5fs	Deletion	Exon 1	–

The affected patients presented with diseases including limb girdle muscular dystrophy 2G (LGMD-2G), dilated and hypertrophic cardiomyopathy (DCM and HCM), intestinal pseudo-obstruction, and telethonin deficiency (Table 5). However, the majority of patients were clinically diagnosed with LGMD-2G (47%) and HCM (29%) (Fig. 3). LGMG-2G patients suffered mostly from symptoms, such as scapular winging**,** contractures, and distal and proximal limb weakness (Table 6). A great number of HCM patients were found to have missense mutations, while LGMG-2G ones showed a high amount of nonsense variants.

**Table 6:** First reported cases due to TCAP genotypes and clinical presentations

Genotype	Protein change	Zygosity	Patient no.	Family	Phenotype	Consanguinity	Ethnicity	Sex	Age on onset	HT	DT	SW	**CT**	FW	CD	Proximal Limb’s weakness	distal Limb’s weakness	Other clinical presentations	References
c.32C > A/c.–178G > T/c.453A > C	p.Ser11Ter/-/p.Ala151 =	Homo/Hetero/Hetero	1	1	**LGMD-2G**	Yes	Indian	M	2 y	+ calves	ND	+	+ ankle	+	ND	ND	ND	mild foot drop, progressive pectoral girdle	[19]
c.32C > A/c.–178G > T/c.453A > C	p.Ser11Ter/-/p.Ala151 =	Homo/homo/homo	1	1	**LGMD-2G**	Yes	Indian	F	8 y	+ calves	ND	+	+ ankle	+	ND	ND	ND	Wheelchair-bound state, mild foot drop, progressive pectoral girdle	[19]
c.32C > A/c.–178G > T/c.453A > C	p.Ser11Ter/-/p.Ala151 =	Homo/homo/homo	1	1	**LGMD-2G**	Yes	Indian	F	8 y	+ calves	ND	+	+ ankle	+	ND	ND	ND	Ambulant, mild foot drop, progressive pectoral girdle	[19]
c.1630G > A	p.Arg18Gln	Hetero	1	2	IDC	ND	Caucasian	ND	ND	ND	ND	ND	ND	ND	+	ND	ND		[20]
c.1968G > A	p.Glu49Lys	Hetero	1	3	IDC	ND	Caucasian	ND	ND	ND	ND	ND	ND	ND	+	ND	ND		[20]
c.2244C > G	p.Pro141Ala	Hetero	1	4	IDC	ND	Caucasian	ND	ND	ND	ND	ND	ND	ND	+	ND	ND		[20]
c.75G > A	p.Trp25Ter	Homo	1	5	**LGMD-2G**	ND	Moldavian	F	15 y	+ calves	ND	ND	ND	ND	ND	+ the lower extremities	+ anterior compartment of legs	mild weakness in shoulder girdle muscles	[21]
c.157C > T	p.Gln53Ter	Homo	2	6	LGMD-2G	ND	Brazilian	ND	ND	ND	ND	ND	ND	ND	ND	ND	ND	ND	[22]
c.637–640delGG/c.157C > T	-/p.Gln53Ter	Com Hetero	1	7	LGMD-2G	ND	Brazilian	ND	ND	ND	ND	ND	ND	ND	ND	ND	ND	ND	[22]
c.172C > T	p.Gln58X	Homo	1	8	telethonin deficiency	No	French	M	9 m	ND	ND	+	+ the Achilles’ tendons	ND	ND	+	ND	delayed motor milestones, waddling gait, mild hyperlordosis, mild upper limb distal joint hyperlaxity, Gower’s maneuver	[23]
c.37–39delGAG	p.Glu13del	Homo	1	9	HCM	No	Caucasian	M	47 y	ND	ND	ND	ND	ND	ND	ND	ND	Dyspnea, (pre)syncope	[6]
c.37–39delGAG	p.Glu13del	Homo	1	10	HCM	No	Caucasian	M	37 y	ND	ND	ND	ND	ND	+	ND	ND	Angina, dyspnea, sudden cardiac death	[6]
c.208C > T	p.R70W	Homo	1	11	HCM	No	Caucasian	F	44 y	ND	ND	ND	ND	ND	+	ND	ND	Asymptomatic, Dyspnea, Atrial fibrillation	[6]
c.269C > T	p.P90L	Homo	1	12	HCM	No	Caucasian	F	26 y	ND	ND	ND	ND	ND	+	ND	ND	Angina, dyspnea, presyncope, Atrial fibrillation	[6]
c. 226C > T	p.Arg76Cys	Hetero	1	13	Intestinal pseudo-obstruction	ND	Italian	M	42 y	ND	ND	ND	ND	ND		ND	ND	intestinal pseudo-obstruction, delayed small bowel and colon transit	[24]
c.244C > T	p.Gln82X	Homo	1	14	LGMD-2G	No	Indian	M	8 y	ND	+ Thigh, calf muscles, gluteal	+	+ asymmetric Achilles tendon, bilateral elbow flexion, hip and ankle joints	+		+ Especially lower	+	toe-walking, frequent fall, a wide-based gait with lumbar hyperlordosis, wheelchair bound, distal phalanx flexion weakness, a few episodes of chocking	[25]
c.255C > A	p.Tyr85*	Homo	1	15	LGMD-2G	Yes	Spanish		2 y	ND	+ Thighs, the tibialis anterior muscles	+	+ symmetric Achilles tendon, patellar	ND		+	+	toe walking, frequent fall, asymmetric calves, Gowers’ maneuver	[26]
c.316C > T	p.Arg106Cys	Hetero	2	16	HCM	No	Danish	ND	30–32 y	ND	ND	ND	ND	ND	+	ND	ND	apical hypertrophy	[17]
c.* + 76G > T		Homo	1	17	HCM	No	Danish	ND	ND	ND	ND	ND	ND	ND		ND	ND	ND	[17]
c.388C > T	p.R130C	Homo	1	18	DCM	ND	British	ND	ND	ND	ND	ND	ND	ND		ND	ND	ND	[27]
c.472C > A	p.R158S	Homo	1	19	DCM	ND	British	ND	ND	ND	ND	ND	ND	ND		ND	ND	ND	[27]
c.493C > G	p.Q165E	Homo	1	20	DCM	ND	British	ND	ND	ND	ND	ND	ND	ND		ND	ND	ND	[27]
c.410C > T	*p.*Thr137Ile	Hetero	1	21	HCM	No	Japanese	F	29 y	ND	ND	ND	ND	ND	+	ND	ND		[10]
c.458G > A	p.R153H	Homo	1	22	HCM	No	Japanese	M	48 y	ND	ND	ND	ND	ND	+	ND	ND		[10]
c.395A > C	p.E132Q	Hetero	1	23	DCM	No	Korean	M	34 y	ND	ND	ND	ND	ND	+	ND	ND	heart failure	[10]
c.472C > A	p.Arg158Ser	Homo	1	24	DCM	No	Finnish	ND	ND	ND	ND	ND	ND	ND	+	ND	ND	LV systolic dysfunction, hypertensive heart disease, primary valve disease, coronary artery disease	[28]
c.472C > T	p.R158C	Homo	1	25	DCM	ND	Canadian	ND	ND	ND	ND	ND	ND	ND	+	ND	ND	Hypertension, diabetes, thyroid conditions	[29]
c.90_91del	p.Ser31HisfsX11	Homo	1	26	LGMD-2G	Yes	Turkish	F	2 y	ND	+ the dorsal thighs and calves	ND	+ Achilles tendon	ND	ND	+ Especially leg	+ Especially legs	tiptoe-walking, myalgia, hyperlordosis,	[30]
c.26_33dupAGGTGTCG	p.Arg12fsTer31	Homo	2	27	LGMD-2G	Yes	Chinese–Cambodian	M	late teenage years	+ Mild calf	ND	+	+ Achilles tendon	ND	ND	ND	ND	progressive muscle weakness, mild weakness of eye closure, mild weakness of most other muscle groups	[31]
c.45_46delTG	p.Cys15Ter	Het	1	28	LGMD-2G	No	Chinese	ND	late childhood	+ calf	ND	+	ND	ND	ND	+	+	wasted sternal head of pectoralis major, finger drop, foot drop	[32]
c.100delC/c.166insG	p.Glu35Argfs*33/p.Gln56Argfs*52	Com hetero	1	29	LGMD-2G	ND	Chinese	ND	ND	ND	ND	ND	ND	ND	+	ND	ND	Vacuolar myopathy	[33]
c.37_39delGAG/c.496_499delAGAG	p.Glu13del /p.Arg166AlafsTer21	Comp hetero	1	30	CMD	ND	Korean	M	2 y	ND	+	ND	ND	ND	ND	ND	ND	muscle fiber necrosis, increased central nuclei and interstitial fibrosis and/or fatty infiltration	[34]
c.171C > G	p.C57W	Hetero	2	31	HCM	No	Portuguese	F/M	45 y	ND	ND	ND	ND	ND	+	ND	ND	paroxysmal atrial fibrillation (AF), a systolic murmur at the left sternal border and aortic area that increased during orthostatism	[35]
c.109_110delGG/c.157C > T	p.Gly37Leufs/p.Gln53X	Comp Hetero	6	32	LGMD-2G	ND	Brazilian	ND	9–15 Y	ND	ND	ND	ND	ND	ND	+ Upper	ND	ND	[22]
c.165–166insG		ND	1	33	LGMG-2	ND	ND	ND	ND	ND	ND	ND	ND	ND	ND	ND	ND	ND	*(ClinVar)
c.110 + 5G > A		ND	1	34	LGMG-2	ND	ND	ND	ND	ND	ND	ND	ND	ND	ND	ND	ND	ND	*(ClinVar)
c.25–31dup	p.Ser11Ter	Homo	1	35	LGMD-2G	ND	ND	ND	26 y	ND	ND	ND	ND	ND	+	ND	ND	muscular dystrophy, myopathy, muscle weakness	*(ClinVar)
c.66G > A	p.Trp22Ter	Homo	1	36	HCM	ND	ND	ND	ND	ND	ND	ND	ND	ND	ND	ND	ND	ND	*(ClinVar)
c.34dup	p.Glu12fs	Homo	1	37	LGMG-2G	ND	Iranian	M	39–40 y	ND	ND	ND	ND	ND	ND	ND	ND	Myopathy	*(ClinVar)
c.43–49dup	p.Arg17delinsLeuTer	Hetero	1	38	HCM	ND	ND	ND	ND	ND	ND	ND	ND	ND	ND	ND	ND	ND	*(ClinVar)
c.103G > T	p.Glu35Ter	Homo	1	39	HCM	ND	ND	ND	ND	ND	ND	ND	ND	ND	ND	ND	ND	ND	*(ClinVar)
c.110_110 + 1del		ND	1	40	LGMG	ND	ND	ND	ND	ND	ND	ND	ND	ND	ND	ND	ND	ND	*(ClinVar)
c.136_137del	p.Gln46fs	ND	1	41	HCM	ND	ND	ND	ND	ND	ND	ND	ND	ND	ND	ND	ND	ND	*(ClinVar)
c.166C > T	p.Gln56Ter	ND	1	42	HCM	ND	ND	ND	ND	ND	ND	ND	ND	ND	ND	ND	ND	ND	*(ClinVar)
c.110 + 1G > A		Homo	1	43	LGMG-2G	ND	ND	ND	ND	ND	ND	ND	ND	ND	ND	ND	ND	Lower limb muscle weakness, Difficulty climbing stairs	*(ClinVar)
c.14–15del	p.Glu5fs	ND	1	44	Abnormality of the musculature	ND	ND	ND	ND	ND	ND	ND	ND	ND	ND	ND	ND	ND	*(ClinVar)

*IDC* Idiopathic dilated cardiomyopathy, *LGMD2G* Limb girdle muscular dystrophy type 2G, *HCM* Hypertrophic cardiomyopathy, *DCM* Dilated cardiomyopathy, *CMD* Congenital muscular dystrophy, *ND* Not defined, *HT* Hypertrophy, *DT* dystrophy, *SW* Scapular Winging, *CT* contractures, *FW* facial weakness, *CD* Cardiac disease *(ClinVar)

## Discussion

This paper was the first to study the *TCAP* gene in the Iranian hypertrophic cardiomyopathies (HCM) and dilated cardiomyopathies (DCM) populations. The cohort consisted of 17 females and 23 males who were clinically diagnosed with HCM and DCM. Their medical records were documented and their blood samples were genetically analyzed, wherein we detected one novel intronic variant c.111-42G > A in intron 1 of the *TCAP* gene in one of our patients. This novel HCM-associated variant was predicted to be polymorphism and have uncertain significance by in-silico analysis.

Dilated and hypertrophic cardiomyopathies are the most frequent cardiac diseases in the affected patients. These disorders impair the myocardium function and lead to severe complications and sudden cardiac death. Various genes were reported to play pivotal roles in presenting cardiomyopathies, such as *MYH7*, *TTN*, *MLP*, and *TCAP*. Pathogenic variants of these genes may disturb the structures of the protein or other proteins that bind them and result in functional alterations of the Z-disc complex. Previous studies on the TCAP gene (telethonin protein) suggested that this gene may be a rare cause of cardiomyopathies among the other involving genes, even though dysfunctional telethonin interferes organizing of the structure of sarcomere assembly and regulates the sarcomere length. Therefore, genetic testing is required for the patients to identify their disease-causing variants and apply efficient treatment for alleviating their symptoms and also detect other susceptible family members before worsening their manifestation.

Hitherto, 44 mutations have been detected in the TCAP that can cause various phenotypes ranging from the most common symptoms of hypertrophy, **s**capular winging, and **c**ontractures to fewer common ones, such as intestinal complications. LGMD-2G and HCM were the top two common diseases among the patients and a great number of them were found to have nonsense and missense variants, respectively.

Mainly due to the lack of studies on the *TCAP* gene mutations in the Iranian population, we investigated the *TCAP* gene in our patients for the presence of HCM and DCM-susceptibility mutations. However, we identified no disease-causing variants in the gene among our cohort suggesting the *TCAP* gene may not be a common cause of heart failure among Iranian patients.

Since our cohort was limited, further analysis is needed to reach a conclusive result regarding the role of *TCAP* gene mutations in Iranian patients with HCM and DCM.

## Data Availability

Upon a reasonable request, additional data are provided from the corresponding author.
